# Ultrasonography as a diagnostic tool for Neural Pain in Leprosy

**DOI:** 10.1371/journal.pntd.0010393

**Published:** 2022-04-29

**Authors:** Clarissa Neves Spitz, Roberto Mogami, Izabela Jardim Rodrigues Pitta, Mariana Andrea Vilas Boas Hacker, Anna Maria Sales, Euzenir Nunes Sarno, Marcia Rodrigues Jardim

**Affiliations:** 1 Post-Graduate Program in Neurology, Federal University of the State of Rio de Janeiro, Rio de Janeiro, Brazil; 2 Leprosy Laboratory, Oswaldo Cruz Institute, Fiocruz, Rio de Janeiro, Brazil; 3 Pedro Ernesto University Hospital/Rio de Janeiro State University, Rio de Janeiro, Brazil; KU Leuven, BELGIUM

## Abstract

Leprosy is still a prevalent disease in Brazil, representing 93% of all occurrences in the Americas. Leprosy neuropathy is one of the most worrying manifestations of the disease. Acute neuropathy usually occurs during reaction episodes and is called neuritis. Twenty-two leprosy patients were included in this study. These patients had neural pain associated with ulnar sensory neuropathy, with or without adjunct motor involvement. The neurological picture began within thirty days of the clinical evaluation. The patients underwent a nerve conduction study and the demyelinating findings confirmed the diagnosis of neuritis. Ultrasonographic study (US) of the ulnar nerve was performed in all patients by a radiologist who was blinded to the clinical or neurophysiological results. Morphological characteristics of the ulnar nerve were analyzed, such as echogenicity, fascicular pattern, transverse cross-sectional area (CSA), aspect of the epineurium, as well as their anatomical relationships. The volume of selected muscles referring to the ulnar nerve, as well as their echogenicity, was also examined. Based on this analysis, patients with increased ulnar nerve CSA associated with loss of fascicular pattern, epineurium hyperechogenicity and presence of power Doppler flow were classified as neuritis. Therefore, patients initially classified by the clinical-electrophysiological criteria were reclassified by the imaging criteria pre-established in this study as with and without neuritis. Loss of fascicular pattern and flow detection on power Doppler showed to be significant morphological features in the detection of neuritis. In 38.5% of patients without clinical or neurophysiological findings of neuritis, US identified power Doppler flow and loss of fascicular pattern. The US is a method of high resolution and portability, and its low cost means that it could be used as an auxiliary tool in the diagnosis of neuritis and its treatment, especially in basic health units.

## Author summary

Leprosy remains a public health problem, despite being an ancient disease. In most cases, it is associated with neuropathy that can be acute or chronic and this neural involvement can occur before, during, or after multidrug therapy (MDT). Acute neuropathy usually occurs during reaction episodes and is called neuritis. It causes compromised nerve function associated with nociceptive pain [1]. The inflammatory process can also develop neuroplasticity of the peripheral and central nervous systems, which perpetuates this sensation; this phenomenon is known as neuropathic pain, a chronic sensation in leprosy. Differential diagnosis between nociceptive and neuropathic pain is not always easy and in some patients there may be an overlap between nociceptive and neuropathic pain. Currently, clinical and electrophysiological criteria are used for the differential diagnosis of neural pain [2], but especially in the case of recurrent neuritis, these criteria are insufficient. Recent knowledge about pain mechanisms does not define the limit as to where nociceptive ends and neuropathic begins, suggesting that this process is continuous, in addition to being associated. Ultrasonography (US) has been used as an auxiliary tool in the early diagnosis of peripheral nervous system diseases. The method can be implemented in addition to clinical and neurophysiological studies for the diagnosis of neuritis, which allows for the early identification and treatment of the disease [3,4].

## Introduction

Leprosy neuropathy can be acute or chronic and neural involvement can occur before, during or after multidrug therapy, especially during reactional episodes, when neuritis occurs [1]. Neuritis causes loss of function and can be irreversible if not properly treated. These episodes are usually accompanied by nociceptive pain resulting from the physiological activation of pain receptors and is related to tissue injury [5]. Neural pain in leprosy can also be due to damage or dysfunction of the somatosensory system, called neuropathic pain; this usually occurs in patients with chronic neural impairment. Some studies have shown that neuritis may be a predisposing factor for neuropathic pain [2]. Therefore, neural pain can be acute, as in the nociceptive pain that accompanies neuritis, or chronically, as in neuropathic pain. However, the differential diagnosis between nociceptive and neuropathic neural pain is not always easy.

Neuritis reflects the immune-mediated inflammatory process that can result in nerve damage if not identified and treated effectively. Clinically, neuritis is characterized by spontaneous or palpation-related pain of the thickened nerve associated with sensory and motor impairment. Neurophysiological findings of demyelination can help support the diagnosis [6–8]. However, the diagnosis of neuritis is sometimes challenging. Some patients have “silent neuritis”, which is a sensory impairment associated or not with motor impairment, but without pain complaint. For this reason, nerve damage goes unnoticed and, if left untreated, it evolves with incapacitating consequences [9]. In addition, recurrent neuritis may occur in patients with neural pain due to chronic neuropathy [7,9–11]. Ultrasonography can provide objective evidence of nerve enlargement based on its measurement. Furthermore, it has already been described in the literature that the presence of power Doppler flow in a peripheral nerve may be an early sign of neuritis [12,13].

The aim of this study was to evaluate the importance of US findings in patients with acute neuropathy in leprosy and to correlate ultrasound changes with clinical and electrophysiological changes in the diagnosis of ulnar neuritis.

## Methods

### Ethics statement

The study was approved by the Research Ethics Committee of the Oswaldo Cruz Institute (03822818.8.0000.5248). All patients voluntarily provided their written informed consent.

### Patient selection

The present study was carried out at the Leprosy Outpatient Clinic of the Oswaldo Cruz Institute, Rio de Janeiro—Brazil, from June 2018 to December 2019. Twenty-two leprosy patients with sensitive ulnar neuropathy, associated or not with concomitant motor impairment, which had started up to thirty days before the evaluation, and which may or may not be associated with neural pain, were included. Patients with diabetes mellitus, alcoholism, rheumatic diseases, renal failure, type I regional complex pain syndrome, fibromyalgia, surgery or ulnar nerve biopsy were excluded. During the period of this study, 450 patients were evaluated at the Leprosy Laboratory, 325 from the Neurology Sector. Of the total of 41 eligible patients, 16 were excluded due to concomitant diseases with neuropathy and another three due to ulnar nerve luxation.

### Clinical evaluation

The patients underwent neurological clinical examination assessment on the face and four limbs according to the protocol of the Leprosy Outpatient Clinic of Oswaldo Cruz Institute [14]. The ulnar nerve was evaluated for the presence of thickening, paresthesia, and neural pain. Tactile sensitivity was tested with Semmes-Weinstein nylon monofilaments and different sensory responses were mapped using values applied to each filament (1 = 300 g, 2 = 4 g, 3 = 2 g, 4 = 0.2 g, and 5 = 0. 05 g) [15,16]. Pain sensitivity assessment was tested with a safety pin, and a refrigerated tuning fork (15°C) was used to assess thermal sensitivity. The muscle strength of the intrinsic muscles of the hands was determined by voluntary muscle testing according to the scale of the Medical Research Council (MRC) [17].

Tendon reflexes were assessed with a Babinski hammer. Finally, vibratory sensitivity was tested with a 128 Hz tuning fork, assigning a score to different levels of perception. Clinical findings of neuritis were considered when neural pain was associated with palpation of the nerve enlargement accompanied by acute sensory or motor deficit. Neuropathic pain was defined when it occurs in the territory of the nerve lesion in question and characterized as spontaneous, continuous or paroxysmal.

Finally, the Portuguese version of the Douleur Neuropathique 4 (DN4) questionnaire was applied [18]. Patients were similarly tested to determine pain intensity visual analogue scale (INT-VAS) using the Likert scale (0 = no symptoms, 10 = worst imaginable sensation).

### Electrophysiologic evaluation

Ulnar nerve sensory and motor nerve conduction studies were measured using the Neuropack μ MEB 9100 EP / EMG measurement system (Nihon Kohden Corp., Tokyo, Japan) in all patients. Skin temperature was measured on the wrists and maintained above 33°C, while room temperature ranged from 29–32°C. Standard methods were performed according to Delisa (1994) [19].

Neurophysiological findings associated with neuritis were the occurrence of a pattern of demyelinating lesion, defined as a reduction in conduction velocity (below 85% of the lower limit of normality) or an extension of the latencies of compound motor action potentials (CMAPs) and sensory nerve action potentials (SNAPs) with slight reduction in amplitude; and presence or absence of demyelination markers, such as conduction block (CB) and temporal dispersion (TD). TD was defined when there was an increase in the duration of CMAPs ≥ 30% from the point of distal stimulation to proximal, and CB when there was a decrease ≥ 50% without TD above 30% [20,21].

### Ultrasound evaluation

A radiologist who was unaware of the clinical and neurophysiological results performed the US. The equipment used was Toshiba Aplio XG model with 12–15 MHz linear transducer and 750 Hz power Doppler parameters, with gain adjustment. Patients were examined in a sitting position with the elbow flexed at 45° and the ulnar nerve was evaluated from the arm to the forearm in the transverse and longitudinal planes [22].

The sectional area of the ulnar nerve was measured in two regions: between the medial epicondyle and the olecranon (epicondylar region) and 2 cm proximal the medial epicondyle (supracondylar region). The sectional area of the ulnar nerve was obtained with freehand delimitation, excluding the epineurium. The authors considered 9.8 mm^2^ the maximum normal value for the ulnar nerve CSA [23]. Ulnar nerve echogenicity was classified as normal or abnormal (being classified as those who had hypoechoic or hyperechoic areas or focal thickening with loss of neural fascicular pattern). Power Doppler was used to assess the occurrence of intrafascicular and perineural vascular flow. Finally, the abductor little finger and ulnar flexor carpal muscles were analyzed for signs of muscle atrophy (hyperechogenicity or loss of volume). The sonographic criteria for neuritis defined in this study were: increase in neural diameter associated with loss of the fascicular pattern and/or epineural thickening, combined with increased vascular flow on power Doppler.

### Statistical analysis

Data were archived in Microsoft Excel and analyzed in OpenEpi. The demographic and clinical characteristics of the sample were described using the median and interquartile range for continuous variables and proportions for categorical variables. Fisher’s and Mann-Whitney exact tests were used to compare the parameters between the evaluated groups. A significance level of 5% was considered.

## Results

Nineteen patients (86%) were male, with a median age of 44 years (range of 18–71). Most had a low level of education, accounting for 86.36% of the sample with less than 11 years of schooling. Fifteen patients (68%) were classified as multibacillary (MB) (Table 1).

**Table 1:** Demographic and clinical characteristics of 22 leprosy patients and neural involvement (2018–2019).

	Variables	n (%)
Sex	Male	19 (86.37)
	Female	3 (13.63)
		
Age	Median (IQR)	44.0 (18–71)
		
Race	White	10 (45.45)
	Afro-descendants	12 (54.45)
	
		
Years of schooling	< 8 years	11 (50.0)
	8–11 years	8 (36.36)
	> 11 years	3 (16.63)
		
WHO classification	PB	7 (31.81)
	MB	15 (68.19)

IQR–interquartile range; PB—paucibacillary; MB—multibacillary.

Predictive clinical neurological signs and symptoms for neuritis were evaluated and included neural thickening, neural pain, paresthesia, and clinical sensory or motor alteration. No clinical features were specific for neuritis in this study. Regarding neural pain, the sensations twinge (37.5%) and shock (31%) were the most frequent, and the mean intensity by the visual analogue scale (INT-VAS) was strong at 8.5 (Table 2).

**Table 2:** Neural pain characteristics (n = 16).

Variables (n%)	n (%)
Quality of pain:	
Twinge	6 (37.5%)
Shock	5 (31%)
Burning	3 (19%)
Hyperesthesia	1 (6%)
Bone pain	1 (6%)
Pain intensity (VAS-INT):	
(0–10) Mean	8.5 (SD:2.02)
Triggering stimulus:	
Spontaneous	4 (25%)
Touch	5 (31%)
Compression	2 (12.5%)
Effort	1 (6%)
At rest	2 (12.5%)
Do not know	2 (12.5%)

SD: standard deviation

**Fig 1:**
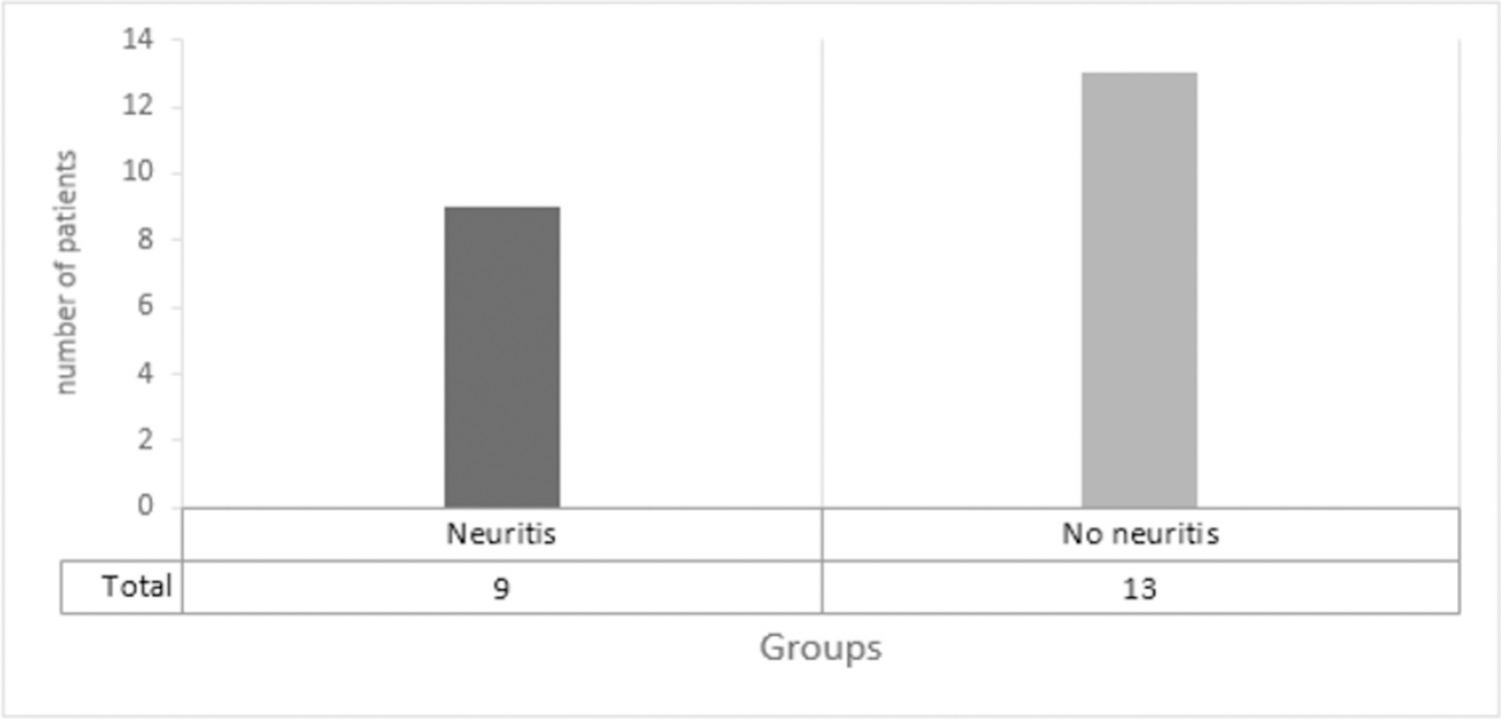
Initial classification of patients with symptoms on the recent ulnar nerve with and without neuritis based on the clinical neurological and electroneuromyography evaluation.

After clinical neurological evaluation, participants were submitted to a nerve conduction study, totaling 22 patients. These were divided into two groups with neuritis (9) and without neuritis (13) based on the clinical-electrophysiological criteria described above, termed the reference criteria (Fig 1).

Subsequently, both groups underwent US and the ultrasound parameters between the two groups, previously divided by the reference criteria (Fig 1), were analyzed. It was noticed that the loss of the fascicular pattern and the presence of flow on power Doppler had a significant association as a indicative of neuritis (p <0.05 for both parameters). On the other hand, epineural thickening and muscle atrophy did not show any significant difference between groups (Table 3). In the group of patients who presented neuritis by the clinical-electrophysiological criteria, the sectional area of the ulnar nerve was significantly increased compared to the group without neuritis, as shown in Table 4.

**Table 3:** Ultrasound variables and their distribution in the two groups of leprosy patients separated by the neuritis reference criteria (neuritis and no neuritis).

Variables	Neuritis (n)	No neuritis (n)	p-value Fisher’s exact test
Loss of fascicular pattern	12	1	<0.001
	
Thickening of epineurium	8	3	0.057
	
Power Doppler	11	0	<0.001
	
Muscle atrophy	4	1	0.248
	

**Table 4:** Values of the area of the ulnar nerve at the level of the medial epicondyle (ulnar groove) and 2 cm proximal to this, between groups with and without neuritis as determined by the reference criteria. Mann-Whitney test.

Groups	Epicondylar level (CSA)		Supracondylar level (CSA)	
With neuritis	Mean	11.0	Mean	12.0
	SD	7.94	SD	12.1
	Median	8.50	Median	6.0
				
Without neuritis	Mean	25.8	Mean	24.0
	SD	18.9	SD	16.3
	Median	20.0	Median	20.5
				
Total	Mean	19.0	Mean	18.0
	SD	16.5	SD	15.2
	Median	12.5	Median	12.0
	p = 0.003		p = 0.050	

CSA–cross-sectional area; SD—standard deviation.

**Fig 2:**
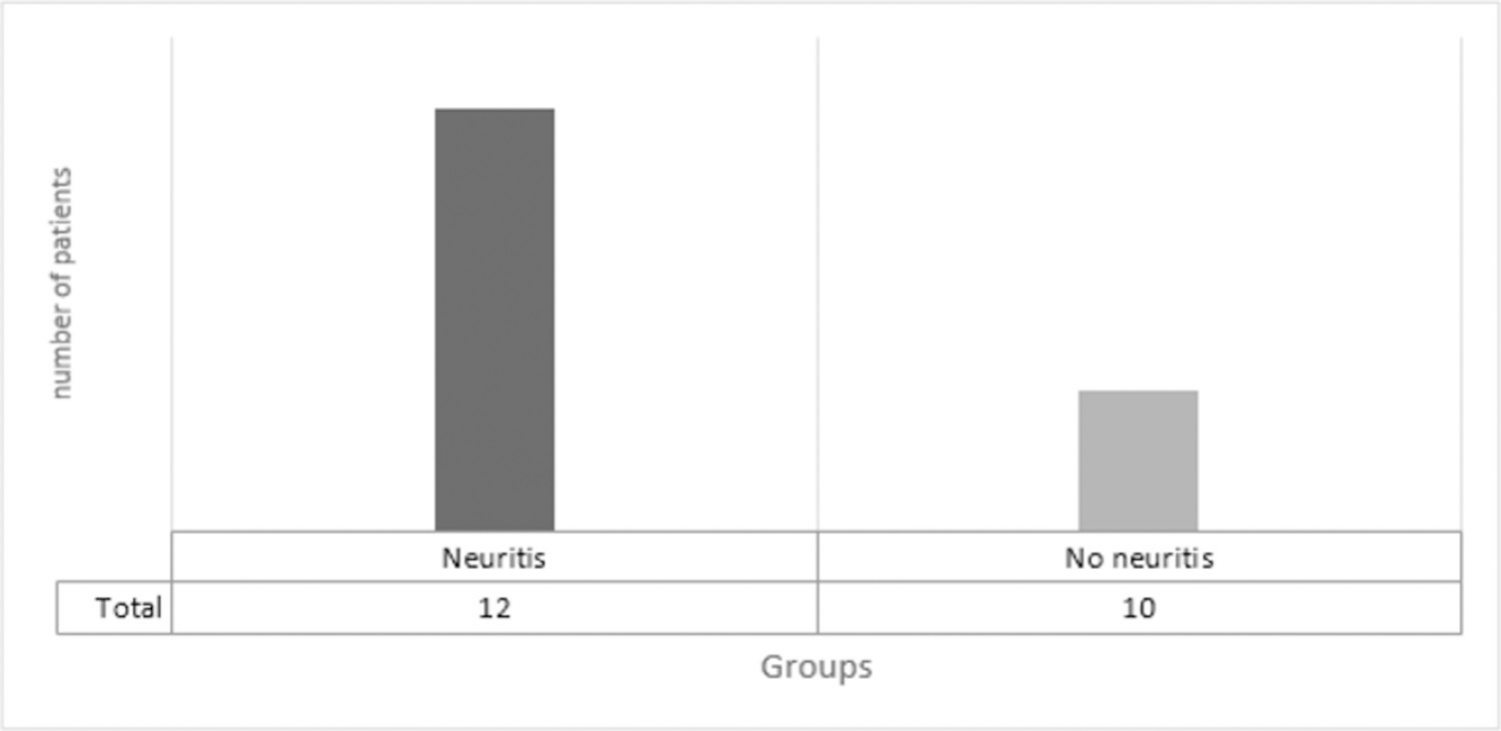
Classification of patients with symptoms in the ulnar nerve in neuritis and without neuritis based on ultrasound assessment.

Therefore, based on the ultrasound criteria, the 22 participants were re-classified into 2 groups with neuritis (12) and without neuritis (10), as shown in Fig 2.

**Fig 3:**
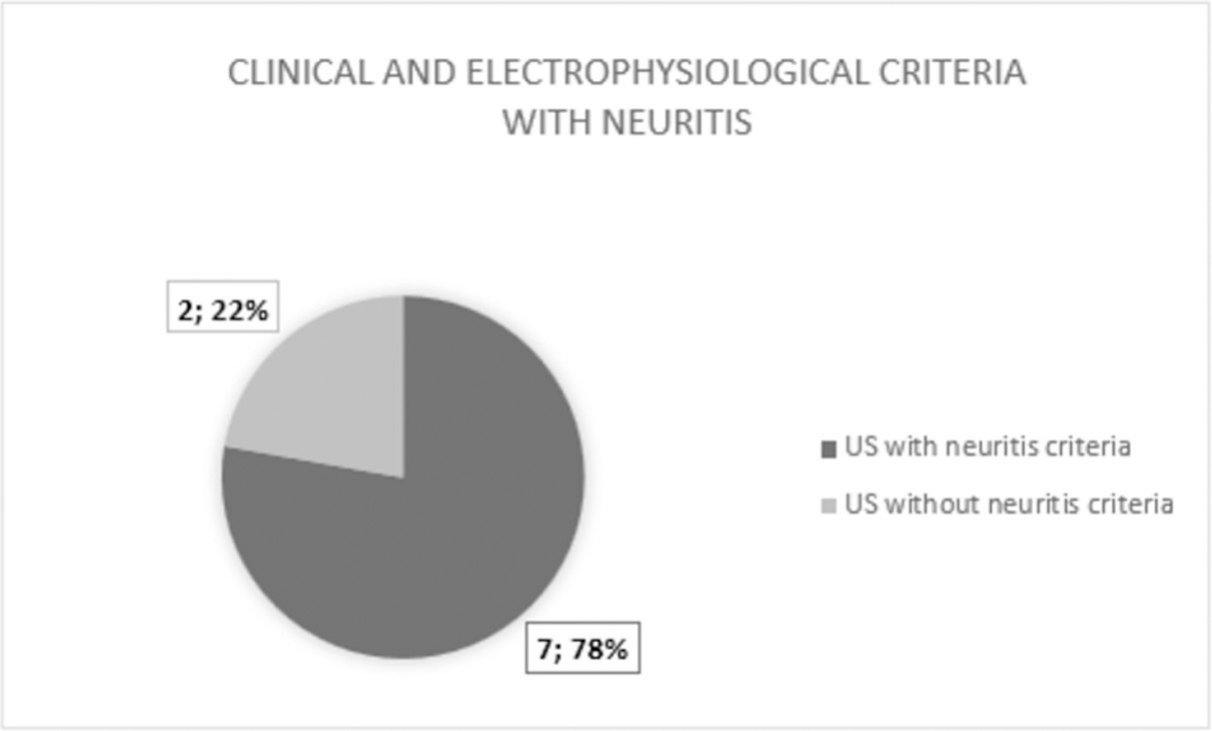
Comparison of patients classified as having neuritis by clinical and electrophysiological criteria (reference criteria) with the ultrasound classification.

**Fig 4:**
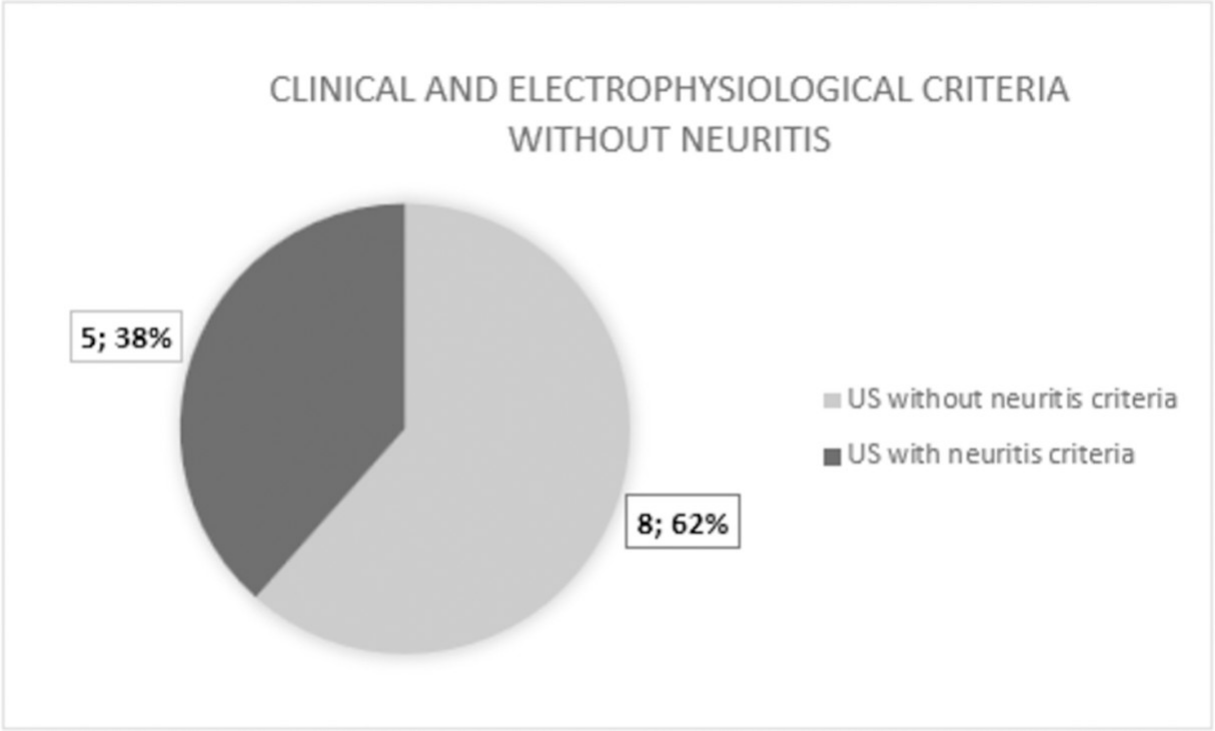
Comparison of patients classified as ´no neuritis´ by clinical and electrophysiological criteria (reference criteria) with the ultrasound classification.

Finally, the correlation between the findings and definition of neuritis was performed based on the reference and ultrasound criteria (Figs 3 and 4). Regarding the 13 patients initially categorized as having no neuritis, five (38.5%) had ultrasound criteria for neuritis. Comparative analysis using Fisher’s test showed a statistically significant diagnostic agreement between the clinical-electrophysiological and US methods for neuritis, with a p-value of 0.04622.

## Discussion

Neuritis events can be recurrent and, after a few episodes of neuritis, there can be a mixture of acute and chronic signs of leprosy neuropathy. This condition makes the diagnosis of neural pain etiology a challenge [7,10,11].

In this study, there was a predominance of patients with the MB form (68%), as described in the literature [2,24]. The higher prevalence of nerve damage in MB patients justifies the higher prevalence of nerve pain in this group of patients [11]. The occurrence of neural pain in leprosy has been systematically reviewed the results obtained vary from 17% to 70.3% among all leprosy patients with neuropathy [25,26]. At the Souza Araújo Leprosy Outpatient Clinic, a reference center in the State of Rio de Janeiro situated in the Oswaldo Cruz Institute, the annual prevalence recorded is 15% [2]. The occurrence of neuritis in the literature can reach 54% [27] and neural thickening—an essential sign of neuritis—does not regress after treatment. Neuritis is described as neural pain associated with sensory impairment with or without motor impairment, related to neural thickening [6–8]. Patients with chronic leprosy neuropathy may experience neuropathic pain [2,10,28,29]. Neurophysiological findings of demyelination have been used to aid in the diagnosis of neuritis. However, in patients with neurological sequelae who present ulnar nerve thickening on physical examination and acute neural pain, the findings may be related to both recurrent neuritis and neuropathic involvement. Differentiating these two conditions by electrophysiology is very difficult [25]. From the group of patients without neuritis according to the reference criteria, five of them had neuritis findings based on the US criteria. These patients had previous chronic neuropathy, with extensive axonal damage, and it was not possible to meet the electrophysiological criteria for demyelination [21]. This condition is particularly important in leprosy, as it is not uncommon to observe the recurrence of neuritis in nerves with pre-existing damage. As discussed above, the reference methods so far are limited for the diagnosis of neuritis superimposed on previous neural damage [30].

Our study showed a positive association (p<0.05) between neuritis according to the reference criteria and loss of the fascicular pattern and presence of flow on power Doppler. Lugão et al. (2016) [12] also found that the detection of intra/perineural flow was a marker of active neuritis and described that vascular flow on power Doppler was directly proportional to the increase in nerve diameter. Detection of intra/perineural flow may be an important finding as a feature of active neuritis.

Our study also showed an association between neuritis and focal thickening of the ulnar nerve, at the epicondylar and supracondylar level in the US, which is in agreement with other studies that found similar results [23,31,32]. Nerve thickening as an isolated finding is not pathognomonic for leprosy neuropathy, therefore, it is essential that there be a set of other associated sonographic changes to make this diagnosis more likely. Hypoechoic focal areas associated with loss of the ulnar nerve fascicular pattern are other findings that can be observed in leprosy-related neuropathy. Elias et al. (2009) [23] observed this change in 81% of the ulnar nerves and Martinoli et al. (2000) [32] found ulnar nerve enlargement associated with fascicular abnormalities in 52% of the nerves. Changes in fascicular architecture have been reported as one of the most relevant findings in neuritis. However, it is unknown whether this is an irreversible finding after nerve damage.

The sample of our study was small, requiring further studies with a larger sample population to confirm the findings. Another prospective study is underway at our center to assess the follow-up of patients with neuritis during and after treatment with corticosteroids. These results may help to better understand the finding of increased blood flow identified by power Doppler in patients with leprosy neuritis.

The present study shows that US can be an auxiliary tool to reduce misdiagnosis of neural pain. Furthermore, as it is a method with high image resolution and low cost, it is suggested as an additional tool. Early detection of nerve involvement can help prevent impairments, as leprosy is the most common infectious cause of neuropathy worldwide. It is hoped that these findings will ensure more reliable diagnoses and facilitate case management in resource-constrained basic health facilities.
